# Dose-Dependent Effects of the *Cimicifuga racemosa* Extract Ze 450 in the Treatment of Climacteric Complaints: A Randomized, Placebo-Controlled Study

**DOI:** 10.1155/2012/260301

**Published:** 2012-12-23

**Authors:** Ruediger Schellenberg, Reinhard Saller, Lorenzo Hess, Jörg Melzer, Christian Zimmermann, Juergen Drewe, Catherine Zahner

**Affiliations:** ^1^Institute for Health Care and Science, 35625 Hüttenberg, Germany; ^2^Institute of Complementary Medicine, University Hospital Zurich (UHZ), 8091 Zurich, Switzerland; ^3^Brunner & Hess, 8038 Zurich, Switzerland; ^4^Clinic for Psychiatry and Psychotherapy, UHZ, 8091 Zurich, Switzerland; ^5^Max Zeller Söhne AG, 8590 Romanshorn, Switzerland

## Abstract

Extracts from *Cimicifuga racemosa* (CR, synonym *Actaea racemosa*) have shown efficacy in trials in women with menopausal symptoms. Yet, dose dependency remains unclear. Therefore, 180 female outpatients with climacteric complaints were treated for 12 weeks in a randomized, double-blind, placebo-controlled, 3-armed trial (CR extract Ze 450 in 6.5 mg or 13.0 mg, or placebo). Primary outcome was the difference in menopausal symptoms (vasomotor, psychological, and somatic), assessed by the Kupperman Menopausal Index between baseline and week 12. Secondary efficacy variables were patients' self-assessments of general quality of life (QoL), responder rates, and safety. Compared to placebo, patients receiving Ze 450 showed a significant reduction in the severity of menopausal symptoms in a dose-dependent manner from baseline to endpoint (mean absolute differences 17.0 (95% CI 14.65–19.35) score points, *P* < 0.0001 for 13.0 mg; mean absolute differences 8.47 (95% CI 5.55–11.39) score points, *P* = 0.0003 for 6.5 mg). QoL and responder rates corresponded with the main endpoint. Changes in menopausal symptoms and QoL were inversely correlated. Reported adverse events and clinical laboratory testing did not raise safety concerns. The CR extract Ze 450 is an effective and well-tolerated nonhormonal alternative to hormone treatment for symptom relief in menopausal women.

## 1. Introduction

On the one hand, menopause is a normal biological process marking the transition of the lives of mature women from a reproductive into a postreproductive phase. On the other hand the profound physiological changes in the peri- and postmenopausal period can provoke complaints. Menopausal changes can lead to vasomotor (e.g., hot flushes, sweating), psychological/vegetative (e.g., insomnia, nervousness/irritability, depressive event, and palpitation), somatic (e.g., joint pain), and urogenital/sexual (e.g., libido changes, dyspareunia, and vaginal dryness) symptoms. They vary in frequency and severity, are related to lifestyle, demographics and sociocultural circumstances, and have been well characterized [1–3]. Hot flushes and night sweating are the cardinal symptoms with highly varying prevalence between 24 to 93% [3–6]. However, the interrelationship of hot flushes and sweating with other neuropsychological symptoms seems to diminish QoL in symptomatic menopausal women [7, 8]. 

Although the role of estrogen appears to be critical and is underlined by the clinical effects of estrogen or estrogen/progestin therapy [9, 10], the mechanisms leading to the development of hot flushes have not been fully elucidated [11]. Since large epidemiological studies with long-term hormonal replacement therapy (HRT), such as the Women's Health Initiative and the Million Women Study [12–15], have shown a small but significantly increased risk for the development of invasive breast cancer, there is an increasing interest in nonhormonal treatment modalities for patients with climacteric symptoms.

*Cimicifuga racemosa* L. (synonym *Actaea racemosa* L., black cohosh) is a perennial medicinal plant native to North America where it has been used for centuries in indigenous medicine for the treatment of various conditions. However, today's sole accepted indications are menopause-related neurovegetative and emotional symptoms. *Cimicifuga racemosa* (CR) extracts are described in a 2003 monograph of the European Scientific Cooperative on Phytotherapy (ESCOP) as a pharmacologically active treatment of climacteric symptoms [16] and in the 2010 community herbal monograph of the Committee on Herbal Medicinal Products of the European Medicines Agency [17]; a well-established use status was granted. 

The precise mechanism of action of CR is controversial, with some studies suggesting that it has no estrogenic effect while others indicate a selective estrogen modulating effect on some tissues, such as bone [18–20]. In addition, serotonin-binding properties in the brain may contribute to its mechanism of action [21, 22]. If indeed CR lacks estrogenic effects, it would have a beneficial influence on climacteric vasomotor and psychiatric symptoms without adversely affecting the development of breast or uterine tissue tumors or increasing the cardiovascular risk. 

Randomized, controlled clinical trials (RCTs) have shown clinically significant effects of extracts from CR [20, 23–26]; but the results have not been consistent in systematic reviews [27–29]. The comparability of the trials is difficult because of differences in dosing, outcome parameters, rating scales, and different CR extracts used [30]. Nevertheless, a meta-analysis performed showed the efficacy of extracts from CR in vasomotor symptoms; but the authors pointed out the heterogeneity of the trials [31].

Dose-dependent effects of an isopropanolic aqueous CR extract have been previously investigated by Liske et al. [25]. Though no placebo or active control treatment was used in this study, both the low- and high-dose improved climacteric symptoms compared to baseline values, and no differences between the treatments could be demonstrated in the Kupperman Menopausal Index (KMI) [32]. For the present study, therefore, placebo treatment was compared to the dose-dependent effects of the CR extract Ze 450 on climacteric symptoms. 

## 2. Patients and Methods

For this multicenter, randomized, double-blind, placebo-controlled, parallel-group trial, 180 female outpatients with menopausal complaints were included. The study took place in four outpatient clinics. Patients were randomized to receive either 13.0 mg Ze 450, 6.5 mg Ze 450 or placebo for 12 weeks. 

The study was performed between March 19, 2002 and July 23, 2003.

### 2.1. Ethics

The study protocol was approved by the Ethics Committee of the Medical Association (Ethik-Kommission Landesärztekammer Hessen), Frankfurt, Germany, and the German Federal Institute for Drugs and Medical Devices (Bundesinstitut für Arzneimittel und Medizinprodukte (BfArM)) was informed prior to its start. Conduct was in accordance with the ICH Guidelines for Good Clinical Practice and the Declaration of Helsinki. A written informed consent was obtained from all patients prior to screening/baseline visit.

### 2.2. Inclusion/Main Exclusion Criteria

Females (age ≥ 40  years) suffering from menopausal syndrome with neurovegetative components, which have been stable anamnestically during the last 2 weeks, and who consulted a physician for the treatment of symptoms were included. The diagnosis of menopausal complaints was confirmed by a Climax Score according to M. Metka and F. H. Fischl. The Climax Score includes neurovegetative, psychical, and atrophic symptoms. By using this tool, the diagnosis of menopausal syndrome was confirmed by the physician. In addition, the baseline Kupperman Menopausal Index (KMI) was recorded by the investigator. Patients were excluded due to previous or current psychological disease that could interfere with their ability to participate in the study; anamnestic or current alcohol or drug abuse; concomitant treatment with psychotropic (in particular benzodiazepines, antidepressants, hypnotics or neuroleptics, tamoxifen, clomifen, and danazol) or hormonally acting drugs such as hormone replacement therapy (HRT); hyperthyroidism; malignant tumors; continuous climacteric bleeding and complaints related to myomas; patients who have taken another experimental drug within a 4-week period prior to the trail; pregnancy/lactation; serious internal disease; previous organ transplantation; premenopausal women with insufficient contraceptive protection; hypersensitivity to one of the ingredients of the trial medication; a body mass index >30.

### 2.3. Study Medication

A 6.5 mg tablet of the (60% v/v) ethanolic CR extract Ze 450 (from rhizomes and roots of CR; drug-extract ratio 4.5–8.5 : 1; Max Zeller Söhne AG, Romanshorn, Switzerland) was used. Placebo was identical looking. 

The treatment schedule for the double-dummy, parallel group design was 2 tablets/once a day given with a meal in the morning: (a) placebo (PLA) + PLA, (b) PLA + 6.5 mg Ze 450, (low dose; LD), and (c) 6.5 mg Ze 450 + 6.5 mg Ze 450 (high dose; HD). Treatment compliance was assessed by a pill count of the returned medication (return of ≤25% was considered compliant).

### 2.4. Outcome Measures

All assessments were done at baseline (visit 1), optionally 6 weeks later (visit 2), and 12 weeks later (visit 3). The severity of menopausal symptoms was assessed at each visit using a modified total KMI score [24, 32–34]. Subitems of this index focused on the neurovegetative symptoms: a 10-item questionnaire of single symptoms whose severity ranged from 0 to 3 (none, mild, moderate, and severe). Score values were multiplied by weighting factors: hot flushes (×4), sweating (×2), insomnia (×2), nervousness/irritability (×2), depressive events/melancholy (×1), vertigo (×1), concentration weakness (×1), joint pain (×1), headache (×1), and palpitations (×1). For this study, the total KMI equalled the sum of the multiplied subitem scores (maximum = 48) and was classified as mild (KMI ≤ 20), moderate (KMI = 21–35), or severe (KMI > 35) [32]. 

The general quality of life (QoL) was assessed by the visual analog scale (VAS). Using a 100 mm printed line, patients checked off how they evaluated their status (0 mm = “best possible quality of life due to health condition”; 100 mm = “worst possible quality of life due to health condition”).

At all visits, routine hematological and clinical-chemical laboratory tests were performed. Thyroid hormones (T3, T4), thyroid-stimulating hormone (TSH), follicle-stimulating (FSH), luteinizing hormone (LH) and estradiol (E2), pregnancy test, and urinalysis were performed only at baseline.

The *primary endpoint* was the difference in the total KMI between both verum groups (HD and LD) and PLA at the end of therapy (week 12), stratified by individual baseline scores in an intention-to-treat (ITT) analysis (patients treated with study medication and with at least one efficacy assessment after baseline). *Secondary endpoints* were (1) the analysis for each subitem of the KMI; (2) analysis of treatment responders (patients with a reduction of ≥50% of the total KMI); (3) a QoL analysis; (4) safety assessment.

### 2.5. Statistical Analysis

Within each clinical site involved the allocation of the randomised treatment was sequential. The random code was supplied by an external provider, using a validated random program (Mathematica, Wolfram Research). Randomization was provided in blocks of six to assure balanced allocation of treatments. Each of the study centres received their own randomization list according to which patients were allocated to the treatment groups. 

Responsible for all statistical aspects, regarding design and analysis of the study, was the Department of Medical Information Technology, University of Giessen, Germany. 

Data management was done by Brunner and Hess, Zurich, Switzerland. Data were entered in duplicate and independently by suitably trained personnel at a statistical institute, who were blinded with respect of treatment group allocation. 

Based on KMI data from previous clinical trials, sample size was conservatively estimated as *n* = 60 patients/group. Assuming an alpha-error = 0.05, mean KMI scores (SD) of 27(15) and 18(15) in the placebo and verum groups, respectively, an anticipated 10% drop-out rate and using the two-sided Mann-Whitney test, a power (1 − *β*) of >90% could be expected (G*Power software, University of Duesseldorf, Germany). A predefined hierarchical statistical design was used. Hierarchically sequenced null hypotheses were that the medians between treatment groups at the end of treatment would be the same, stratified by individual baseline scores: H01 : PLA versus HD; H02 : PLA versus LD; H03 : LD versus HD. The respective alternative hypotheses (HA1 to HA3) stated that the medians would be different. Comparisons were performed by the stratified non-parametric Wilcoxon-Mann-Whitney test (*α* = 0.05, 2-sided, StatXact, Version 9) to test the hypotheses with the following hierarchical-sequentially rejecting procedure: Step 1: H01 versus HA1; Step 2: H02 versus HA2; Step 3: H03 versus HA3. The test procedure was terminated once a null hypothesis could not be rejected. This hierarchical statistical design was used as a method to control type I error for multiple comparisons. Therefore, there was no need to adjust *P* values for multiple comparisons for the 3-step testing. Responder analysis (for total KMI) was done using the same hierarchical sequence by Pearson's Chi-square test. For patients terminating participation prematurely at the interim visit, all available efficacy data were treated according to the principle of last observation carried forward (LOCF).

## 3. Results

**Figure 1:**
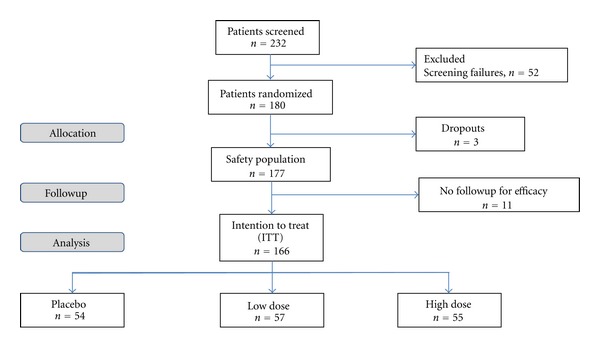
CONSORT diagram of the disposition of participants.

Of the 232 patients originally referred to the clinics, 52 failed the selection criteria. A total of 180 patients were randomized. Their mean age was 51.7 years and mean BMI (Body Mass Index) was 25.2 kg/m^2^ when entering the study. Safety and ITT populations comprised *n* = 177 and *n* = 166 patients, respectively (Figure 1). The majority of patients in the ITT population (*n* = 85, 51.2%) were in the early postmenopausal stage (less than 5 years since last menstruation); fewer patients (*n* = 43, 25.9%) were in the late postmenopausal (more than 5 years since last menstruation) or premenopausal stage (*n* = 38, 22.9%). A similar categorization was obtained when threshold FSH concentration of 40 mIU/mL was used. Smoking habits did not differ between the treatment groups (Table 1). Severity of symptoms (total KMI) ranged from mild (*n* = 29, 17.5%) to moderate (*n* = 109, 65.7%) to severe (*n* = 28, 16.9%). There were no significant differences between the treatment groups in any of the demographic parameters or for baseline levels of T3, T4, TSH, FSH, LH, and E2. 

**Figure 2:**
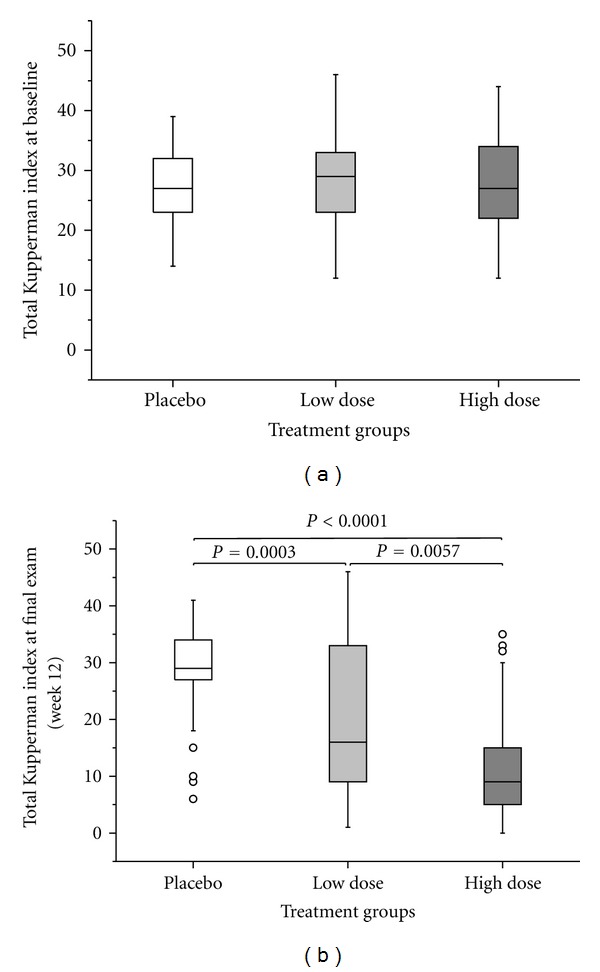
Total KMI (a) at baseline and (b) after 12 weeks of treatment (ITT population, *n* = 166) with PLA, LD, and HD. Circles denote outliers. Two-sided Mann-Whitney test stratified to baseline scores.

Concerning the primary endpoint verum was superior to placebo in reducing the total KMI score in a dose-dependent manner (Table 2, Figure 2). Regarding KMI sub-items, a significant reduction in each item was seen only with the HD group. The clinical relevance was the strongest for vasomotor subitems (e.g., hot flushes, sweating), less for psychological/vegetative sub-items (e.g., insomnia, nervousness/irritability, and depressive events/melancholy) and the smallest for somatic symptoms (joint pain), although significant (Table 3).

Treatment effect size at the end of study (week 12) was dependent on the baseline symptom severity (Table 4). For patients with initially mild (total KMI ≤ 20) and moderate symptoms (20 < total KMI ≤ 35), average KMI scores decreased from baseline values significantly and in a dose-dependent manner by 5.4 (8.3 SD) and 9.6 (11.5 SD) score points in the LD group and by 10.5 (4.4 SD) and 17.8 (8.6 SD) score points in the HD group. This contrasts to the average KMI scores in the PLA group that even increased from baseline values by 12.2 (4.7 SD) (initially mild symptoms) and 0.6 (7.8 SD) (initially moderate symptoms) score points. For patients with initially severe symptoms (total KMI > 35), average scores decreased by 4.8 (8.5 SD) (PLA), 5.8 (12.0 SD) (LD), and 20.1 (9.5) (HD) score points. However, only for the HD group could a significant difference versus the PLA group be demonstrated. 

For premenopausal patients, only the HD showed a significant (*P* < 0.001) decrease in average total KMI scores compared to the PLA group (20.9 (7.6 SD) versus 1.1 (7.8 SD) score points, resp.). In patients in the early and late postmenopausal states, comparable magnitudes of effects of Ze 450 treatments were observed: LD and HD treatments in early postmenopausal women decreased KMI by 11.0 (11.2 SD) and 17.2 (9.7 SD) score points, respectively, whereas PLA increased KMI by 3.3 (7.9 SD) score points. In late postmenopausal women, LD and HD treatment decreased KMI by 10.2 (11.2 SD) and 13.6 (5.8 SD) score points, respectively, whereas PLA increased KMI by 1.5 (12.1 SD) score points. However, in contrast to the early postmenopausal subgroup, in the late postmenopausal subgroup no superiority for the HD over the LD group could be established.

With respect to the secondary endpoints, the responder rate for ≥50% reduction in total KMI increased from 7.4% in the PLA group to 40.4% in the LD group (*P* < 0.001) and increased further to 69.1% in the HD group. The latter was significantly higher than in the PLA group (*P* < 0.001) or the LD group (*P* = 0.002).

**Figure 3:**
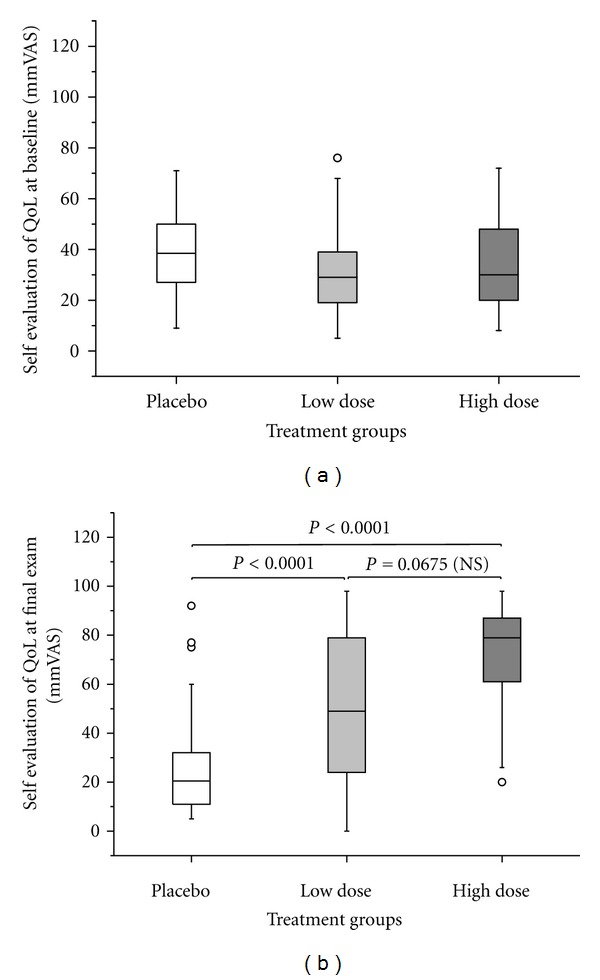
QoL assessment by VAS (a) at baseline and (b) after 12 weeks of treatment (ITT population, *n* = 166) with PLA, LD, and HD. Circles denote outliers. Two-sided Mann-Whitney test stratified to baseline scores.

Corresponding to the reduction in symptom severity, the QoL VAS increased dose-dependently (Figure 3). Changes in menopausal symptoms and QoL were inversely correlated.

As to safety no serious, but 21 nonserious adverse events (AE) occurred in 20 patients: 9 of which were possibly treatment related (5 PLA, 2 LD, and 2 HD group), five were unlikely or not related to the study medication, and the relationship was assessed as unknown for seven. Among the nine possibly study-related AEs, five were of a gastrointestinal nature, a known AE of Ze 450. The frequency of possibly related AEs was higher in the placebo group. Laboratory assessments revealed no clinically significant changes, except for three patients (one from each group) with elevated liver enzyme values. Two were likely caused by excessive alcohol consumption, while the third remained undefined. These three AEs were not clustered in one specific treatment group; but they were equally distributed among the three groups. No safety concerns were raised based on the monitoring of vital signs, physical examination, and laboratory values from the beginning to the end of the study.

## 4. Discussion

Our study demonstrated that the CR extract administered for 12 weeks decreased significantly, and in a dose-dependent manner, the severity of climacteric symptoms in the total KMI. This was predominantly seen in single subitems especially for vasomotor as well as for some psychological symptoms. Furthermore, the administration of CR extract over 12 weeks improved general QoL and was safe. 

The strengths of the current trial are as follows. (a) It analysed one of the unanswered questions in the nonhormonal treatment of menopausal complaints with extracts from CR—the dose-dependency. This was done in a suitable methodological manner with the high internal validity of the 3-arm RCT. Blinding was achieved by applying a double-blind, double-dummy setting in order to avoid any unblinding bias. 

(b) A subgroup analysis showed that both the LD and HD demonstrated, in a dose-dependent manner, a significantly larger effect than placebo in patients with mild and moderate symptom severity. For patients with severe symptoms, however, only the high dose (13.0 mg) of Ze 450 was effective. Compared to the placebo and LD groups, only the high dose reduced significantly the symptom score in patients at all menopausal stages; the LD was superior to placebo only in the early and late postmenopausal stage.

Nonetheless, there are some limitations. (a) Although the study duration was 3 months, a longer duration of 6–12 months would better meet the necessary long-term treatment of most women. This will be the subject of a future trial. (b) The question which menopause scale should be administered is an ongoing discussion. Although the comparison of the Menopause Rating Scale (MRS) with the KMI produced a high correlation of raw scores, the MRS, especially in its second version (MRS-II), is somehow favoured today [35, 36]. This might be due to its better appropriateness for factor analysis of symptom clusters and QoL measurements. Nonetheless, it is known that CR extracts are most effective for vasomotor symptoms therefore it seemed appropriate to use the KMI [28, 31]. Additionally, we tried to overcome this shortcoming with analysing the single symptoms next to total KMI score, giving a subgroup analysis and rating general QoL with the VAS.

The placebo response might have been low in the present study. However, also in other clinical trials in postmenopausal women, with a small number of visits, no reduction of the KMI was observed with placebo treatment [36, 37] as it was the case in the present study. On the other hand, higher placebo effects were observed in trials, where more patients visits had been performed (such as in [34]). This suggests that the study design might influence the placebo response.

The results of our current study partly confirm previous studies with other CR extracts [20, 23–26]. The age and BMI distribution of the patients are comparable to several previously conducted clinical studies with extracts from CR. Nevertheless, the study population is still small and may give predictions at best for middle-European women without heavy overweight. However, Liske et al. [25] have shown that two doses of an isopropanolic aqueous CR extract given for 24 weeks reduced significantly the symptom severity as assessed by KMI to a similar extent. In both treatment groups, total KMI decreased by about 50% from baseline scores of 30.5 (low dose) and 31 (high dose) to scores less than 15 in 70% and 72% of the patients, respectively. The responder rates were comparable to our study, where the rate of patients with at least a 50% decrease in total KMI was 69.1% in the HD group. In contrast to the study of Liske et al., however, we could clearly demonstrate for the first time a dose-dependent effect of a CR extract. The efficacy of other CR extracts was further shown by Osmers et al. [23] using a fixed dose of an isopropanolic extract and a placebo treatment during a 12-week period. In a further study of the same extract continued for 52 weeks, the effect on the number and intensity of hot flushes was confirmed [38] and, in addition, overall tolerability and endometrial safety were demonstrated. On the other hand, after a short-term, 4-week treatment in patients suffering from breast cancer, a similar CR extract demonstrated no superior effect when compared to placebo [39]. However, the extract used in the latter study was not sufficiently characterized and the duration of treatment was shorter than in previous studies [20, 23–26] that have demonstrated a significant reduction in the number and intensity of hot flushes. Additionally, the dose-effect relationship was not investigated. This underscores the difficulties in comparing clinical results between studies using different extracts with potentially different spectra of pharmacologically active constituents.

Whether the treatment effects of Ze 450 can be maintained for longer treatment periods is a subject for further research. Reported AEs did not raise safety concerns.

In conclusion, the CR extract, Ze 450, appears to be an effective and well-tolerated nonhormonal alternative to HRT for symptom relief in menopausal women.

## Figures and Tables

**Table 1:** Demographic details: mean (SD), ITT population.

	*n *	Placebo	*n *	Low dose	*n *	High dose	*P* value^1^
Age (years)	54	50.5 (7.0)	57	52.0 (6.3)	55	52.8 (6.0)	0.168
Weight (kg)	54	67.7 (11.6)	57	70.5 (13.0)	55	68.3 (12.2)	0.433
BMI (kg/m^2^)	54	24.9 (4.3)	57	25.6 (5.1)	55	25.0 (3.9)	0.634
Height (cm)	54	164.9 (5.1)	57	166.3 (7.1)	55	165.2 (7.4)	0.537
KMI (points)	54	27.3 (6.5)	57	28.1 (6.9)	55	28.4 (8.0)	0.692
QoL (mm)	54	37.2 (15.6)	57	31.2 (15.9)	55	34.7 (17.6)	0.150
Premenopausal (*n*)	54	15	57	12	55	11	0.803^2^
Early postmenopausal (*n*)	54	27	57	28	55	30	
Late postmenopausal (*n*)	54	12	57	17	55	14	
Baseline FSH ≤ 40 mIU/mL (*n*)	54	21	57	22	55	15	0.333^2^
Baseline FSH > 40 mIU/mL (*n*)	54	22	57	26	55	33	
Baseline FSH unknown	54	11	57	9	55	7	
Baseline KMI ≤ 20	54	9	57	10	55	10	0.454^2^
Baseline KMI 21–35	54	36	57	41	55	32	
Baseline KMI > 35	54	9	57	6	55	13	
Nonsmoker (*n*)	54	41	57	44	55	39	0.575^3^
Occasional smoker (*n*)	54	3	57	0	55	4	
Moderate smoker (*n*)	54	6	57	7	55	7	
Heavy smoker (*n*)	54	4	57	6	55	5	

^1^Analysis of variance, ^2^Chi-square test, ^3^Fisher's exact test.

**Table 2:** Intention to treat analysis: total KMI after 12 weeks of treatment (*n* = 166).

Treatment	*n *	Baseline		End of study		Absolute differences		Hierarchical test procedure*		
		Mean (SD)	95% CI	Mean (SD)	95% CI	Mean (SD)	95% CI	Step 1	Step 2	Step 3
Placebo	54	27.30 (6.5)	25.53–29.06	28.94 (7.6)	26.87–31.02	1.65 (9.0)	−0.80–4.10	Reference	Reference	
Low dose	57	28.12 (6.9)	26.28–29.96	19.65 (13.1)	16.17–23.13	−8.47 (11.0)	−11.39 to −5.55		*P* = 0.0003	Reference
High dose	55	28.44 (8.0)	26.28–30.60	11.44 (9.1)	8.98–13.90	−17.00 (8.7)	−19.35 to −14.65	*P* < 0.0001		*P* = 0.0057

*According to two-sided Mann-Whitney test stratified to individual baseline scores; 95% CI: 95% confidence interval; SD: standard deviation; *n*: number of patients.

**Table 3:** Change in symptom severity of KMI sub-items after treatment during 12 weeks (ITT population).

Subitem	Treatment group	Baseline			Final examination (12 weeks)			Absolute differences			Hierarchial test procedure*		
		Valid *n *	Mean (SD)	95% CI	Valid *N *	Mean (SD)	95% CI	Valid *N *	Mean (SD)	95% CI	Step 1	Step 2	Step 3
	Placebo	54	8.37 (3.83)	7.33–9.42	54	9.93 (3.27)	9.03–10.82	54	1.56 (3.60)	0.57–2.54	Ref.	Ref.	
Hot flushes	Low dose	57	8.56 (3.25)	7.70–9.42	57	6.32 (4.34)	5.16–7.47	57	−2.25 (4.47)	−3.43 to −1.06		*P* < 0.0001	Ref.
	High dose	55	8.65 (3.83)	7.62–9.69	55	3.20 (3.48)	2.26–4.14	55	−5.45 (4.17)	−6.58 to −4.33	*P* < 0.0001		*P* = 0.0001

	Placebo	54	3.85 (2.05)	3.29–4.41	54	4.70 (1.61)	4.26–5.14	54	0.85 (2.08)	0.28–1.42	Ref.	Ref.	
Sweating	Low dose	57	4.14 (1.77)	3.67–4.61	57	2.74 (2.26)	2.14–3.34	57	−1.40 (2.20)	−1.99 to −0.82		*P* < 0.0001	Ref.
	High dose	55	4.18 (1.82)	3.69–4.67	55	1.60 (1.70)	1.14–2.06	55	−2.58 (2.02)	−3.13 to −2.03	*P* < 0.0001		*P* = 0.0064

	Placebo	54	4.00 (1.90)	3.48–4.52	54	4.19 (1.87)	3.67–4.70	54	0.19 (2.31)	−0.44–0.81	Ref.	Ref.	
Insomnia	Low dose	57	4.04 (1.98)	3.51–4.56	57	2.91 (2.27)	2.31–3.51	57	−1.12 (2.11)	−1.68 to −0.56		*P* = 0.0015	Ref.
	High dose	55	3.93 (2.07)	3.37–4.49	55	1.75 (1.81)	1.26–2.23	55	−2.18 (2.32)	−2.81 to −1.56	*P* < 0.0001		*P* = 0.0044

	Placebo	54	4.11 (1.71)	3.64–4.58	54	4.00 (2.02)	3.45–4.55	54	−0.11 (2.11)	−0.69–0.46	Ref.	Ref.	
Nervousness, irritability	Low dose	57	4.14 (1.64)	3.70–4.58	57	2.70 (2.12)	2.14–3.26	57	−1.44 (1.96)	−1.96 to −0.92		*P* = 0.0005	Ref.
	High dose	55	3.67 (2.03)	3.12–4.22	55	1.64 (1.54)	1.22–2.05	55	−2.04 (2.13)	−2.61 to −1.46	*P* < 0.0001		*P* = 0.0100

Depressive events, melancholy	Placebo	54	1.59 (1.11)	1.29–1.89	54	1.54 (0.97)	1.27–1.80	54	−0.06 (1.11)	−0.36–0.25	Ref.	Ref.	
	Low dose	57	1.77 (1.09)	1.48–2.06	57	1.26 (1.09)	0.97–1.55	57	−0.51 (0.98)	−0.77 to −0.25		*P* = 0.0308	Ref.
	High dose	55	1.80 (1.06)	1.51–2.09	55	0.71 (0.76)	0.50–0.92	55	−1.09 (1.02)	−1.37 to −0.81	*P* < 0.0001		*P* = 0.0020

	Placebo	54	0.67 (0.89)	0.42–0.91	54	0.74 (0.87)	0.50–0.98	54	0.07 (1.01)	−0.20–0.35	Ref.	Ref.	
Vertigo	Low dose	57	0.65 (0.86)	0.42–0.88	57	0.49 (0.83)	0.27–0.71	57	−0.16 (0.56)	−0.31 to −0.01		*P* = 0.0390	Ref.
	High dose	55	0.84 (0.94)	0.58–1.09	55	0.42 (0.63)	0.25–0.59	55	−0.42 (0.81)	−0.64 to −0.20	*P* = 0.0103		NS

	Placebo	54	1.31 (0.97)	1.05–1.58	54	1.02 (1.00)	0.75–1.29	54	−0.30 (1.02)	−0.58 to −0.02	Ref.	Ref.	
Concentration weakness	Low dose	57	1.42 (1.03)	1.15–1.70	57	1.02 (1.09)	0.73–1.31	57	−0.40 (0.90)	−0.64 to −0.16		NS	Ref.
	High dose	55	1.56 (0.94)	1.31–1.82	55	0.76 (0.86)	0.53–1.00	55	−0.80 (0.91)	−1.05 to −0.55	*P* = 0.0350		NS**

	Placebo	54	1.26 (1.17)	0.94 (1.58)	54	1.06 (1.14)	0.74–1.37	54	−0.20 (0.94)	−0.46–0.05	Ref.	Ref.	
Joint pain	Low dose	57	1.49 (1.20)	1.17–1.81	57	1.04 (1.20)	0.72–1.35	57	−0.46 (0.95)	−0.71 to −0.21		NS	Ref.
	High dose	55	1.53 (1.18)	1.21–1.85	55	0.58 (0.81)	0.36–0.80	55	−0.95 (0.95)	−1.20 to −0.69	*P* = 0.0006		*P* = 0.0191**

	Placebo	54	0.89 (0.92)	0.64–1.14	54	0.93 (0.80)	0.71–1.14	54	0.04 (1.05)	−0.25–0.32	Ref.	Ref.	
Headache	Low dose	57	0.81 (0.91)	0.56–1.05	57	0.54 (0.85)	0.32–0.77	57	−0.26 (0.61)	−0.43 to −0.10		*P* = 0.0030	Ref.
	High dose	55	0.96 (0.96)	0.70–1.22	55	0.38 (0.56)	0.23–0.53	55	−0.58 (0.85)	−0.81 to −0.35	*P* < 0.0001		NS

	Placebo	54	1.24 (0.80)	1.02–1.46	54	0.85 (0.90)	0.61–1.10	54	−0.39 (0.83)	−0.62 to −0.16	Ref.	Ref.	
Palpitations	Low dose	57	1.11 (0.90)	0.87–1.34	57	0.63 (0.88)	0.40–0.86	57	−0.47 (0.93)	−0.72 to −0.23		NS	Ref.
	High dose	55	1.31 (1.10)	1.01–1.61	55	0.40 (0.68)	0.22–0.58	55	−0.91 (0.99)	−1.18 to −0.64	*P* = 0.0005		NS**

*Two-sided Mann-Whitney test stratified to baseline values; Ref.: reference.

**Not applicable due to hierarchical design; NS: not significant; 95% CI: 95% confidence interval; *n*: number of patients.

**Table 4:** Change in symptom severity as assessed by total KMI in patient subgroups.

Treatment	*n *	Baseline	End of study	Absolute differences		Hierarchical test procedure*		
		Mean (SD)	Mean	Mean (SD)	95% CI	Step 1	Step 2	Step 3
Baseline KMI ≤ 20								
Placebo	9	17.7 (2.2)	29.9 (4.3)	12.2 (4.7)	8.58–15.86	Ref.	Ref.	
Low dose	10	18.4 (2.7)	13.0 (8.5)	−5.4 (8.3)	−11.35 to −0.55		*P* < 0.001	Ref.
High dose	10	17.3 (3.0)	6.8 (5.0)	−10.5 (4.4)	−13.65 to −7.35	*P* < 0.001		NS
21 ≤ baseline KMI ≤ 35								
Placebo	36	27.2 (3.6)	27.8 (8.1)	0.6 (7.8)	−2.01–3.23	Ref.	Ref.	
Low dose	41	28.7 (4.1)	19.1 (12.6)	−9.6 (11.5)	−13.23 to −5.99		*P* < 0.001	Ref.
High dose	32	27.5 (4.0)	9.7 (7.6)	−17.8 (8.6)	−20.87 to −14.70	*P* < 0.001		*P* = 0.004
Baseline KMI > 35								
Placebo	9	37.2 (1.1)	32.4 (7.7)	−4.8 (8.5)	−11.27–1.72	Ref.	Ref.	
Low dose	6	40.5 (3.5)	34.7 (13.0)	−5.8 (12.0)	−18.38–6.71		NS	Ref.
High dose	13	39.3 (2.4)	19.2 (10.7)	−20.1 (9.5)	−25.83 to −14.33	*P* = 0.001		*P* = 0.022**
Premenopausal								
Placebo	15	29.5 (6.4)	28.4 (8.9)	−1.1 (7.8)	−5.43–3.16	Ref.	Ref.	
Low dose	12	25.5 (8.6)	25.3 (12.3)	−0.2 (5.4)	−3.62–3.29		NS	Ref.
High dose	11	27.6 (9.5)	6.7 (6.3)	−20.9 (7.6)	−26.00 to −15.82	*P* < 0.001		*P* = 0.001**
Early postmenopausal								
Placebo	27	26.4 (6.8)	29.6 (6.7)	3.3 (7.9)	0.13–6.39	Ref.	Ref.	
Low dose	28	27.5 (6.0)	16.6 (12.7)	−11.0 (11.2)	−15.31 to −6.62		*P* < 0.001	Ref.
High dose	30	28.4 (7.2)	11.2 (9.0)	−17.2 (9.7)	−20.81 to −13.53	*P* < 0.001		*P* = 0.039
Late postmenopausal								
Placebo	12	26.6 (5.6)	28.1 (8.3)	1.5 (12.1)	−6.21–9.21	Ref.	Ref.	
Low dose	17	30.9 (6.5)	20.7 (13.6)	−10.2 (11.2)	−15.99 to −4.48		*P* = 0.006	Ref.
High dose	14	29.2 (8.9)	15.6 (9.8)	−13.6 (5.8)	−16.91 to −10.24	*P* = 0.001		NS

*Two-sided Mann-Whitney test stratified to baseline values; Ref.: reference; descriptive *P* values.

**Not applicable due to hierarchical design; NS: not significant; SD: standard deviation; 95% CI: 95% confidence interval; *n*: number of patients.
